# Network analysis of depressive symptoms in Hong Kong residents during the COVID-19 pandemic

**DOI:** 10.1038/s41398-021-01543-z

**Published:** 2021-09-06

**Authors:** Teris Cheung, Yu Jin, Simon Lam, Zhaohui Su, Brian J. Hall, Yu-Tao Xiang, the International Research Collaboration on COVID-19, Lorna Kwai Ping Suen, Shun Chan, Hilda Sze Wing HO, Kin Bong Hubert Lam, Emma Yun-zhi Huang, Ying Xiao, Fernanda Maria Vieira Pereira-Ávila, Elucir Gir, Menevse Yildirim, Seyda Seren Intepeler, Tella Lantta, Kyungmi Lee, Nayeon Shin, Laurence Lloyd Parial, Tor Michael Rossing, Ching Yuk Hon, Merissa Tsang, Jessica P. Braz Poeys, Tommy Kwan Hin Fong

**Affiliations:** 1 School of Nursing, Hong Kong Polytechnic University, Hong Kong SAR, China; 2 College of Education for the Future, Beijing Normal University, Beijing, China; 3 Center on Smart and Connected Health Technologies, Mays Cancer Center, School of Nursing, UT Health San Antonio, San Antonio, TX USA; 4 Global and Community Mental Health Research Group, New York University (Shanghai), Shanghai, China; 5 School of Global Public Health, New York University, New York, NY USA; 6 Unit of Psychiatry, Department of Public Health and Medicinal Administration, Institute of Translational Medicine, Faculty of Health Sciences, University of Macau, Macao SAR, China; 7 Centre for Cognitive and Brain Sciences, University of Macau, Macao SAR, China; 8 Institute of Advanced Studies in Humanities and Social Sciences, University of Macau, Macao SAR, China; 9 School of Nursing, Tung Wah College, Hong Kong SAR, China; 10 Squina International Centre for Infection Control, School of Nursing, The Hong Kong Polytechnic University, Hong Kong SAR, China; 11 Department of Psychology, York University, Toronto, Canada; 12 Nuffield Department of Population Health, University of Oxford, Oxford, UK; 13 Department of Social Worker, Zhongshan Polytechnic, Guangdong, China; 14 Faculty of Medicine, Macau University of Science and Technology, Macao, Macao; 15 Fluminense Federal University, Rio das Ostras, Brazil; 16 University of São Paulo, School of Nursing at Ribeirão Preto, Ribeirão Preto, Brazil; 17 Department of Nursing Management, Fethiye Faculty of Health Sciences, Muğla Sıtkı Kocman University, Mugla, Turkey; 18 Department of Nursing Management, Faculty of Nursing, Dokuz Eylul University, Izmir, Turkey; 19 Department of Nursing Science, University of Turku, Turku, Finland; 20 Samsung Medical Center, Seoul, Korea; 21 CHA University, Bundang CHA Medical Center, Seongnam, Korea; 22 School of Nursing, The Hong Kong Polytechnic University, Hong Kong, China; 23 SAG Flowmedik Oy, Helsinki, Finland; 24 Agape Acupuncture Clinic, San Mateo, CA USA; 25 Westways Staffing Inc, Austin, TX USA; 26 School of Nursing, The Hong Kong Polytechnic University, Hong Kong SAR, China

**Keywords:** Depression, Scientific community

## Abstract

In network theory depression is conceptualized as a complex network of individual symptoms that influence each other, and central symptoms in the network have the greatest impact on other symptoms. Clinical features of depression are largely determined by sociocultural context. No previous study examined the network structure of depressive symptoms in Hong Kong residents. The aim of this study was to characterize the depressive symptom network structure in a community adult sample in Hong Kong during the COVID-19 pandemic. A total of 11,072 participants were recruited between 24 March and 20 April 2020. Depressive symptoms were measured using the Patient Health Questionnaire-9. The network structure of depressive symptoms was characterized, and indices of “strength”, “betweenness”, and “closeness” were used to identify symptoms central to the network. Network stability was examined using a case-dropping bootstrap procedure. Guilt, Sad Mood, and Energy symptoms had the highest centrality values. In contrast, Concentration, Suicide, and Sleep had lower centrality values. There were no significant differences in network global strength (*p* = 0.259), distribution of edge weights (p = 0.73) and individual edge weights (all *p* values > 0.05 after Holm–Bonferroni corrections) between males and females. Guilt, Sad Mood, and Energy symptoms were central in the depressive symptom network. These central symptoms may be targets for focused treatments and future psychological and neurobiological research to gain novel insight into depression.

## Introduction

Depression is a common psychiatric problem characterized by a range of symptoms such as low mood, guilt, and worthlessness [1]. Individuals with depression may experience immense personal and familial suffering, and they may also have other adverse outcomes such as impaired functioning, insomnia, economic burdens and even suicide [2–5]. In order to reduce the risk of depression and provide timely and effective treatments, it is important to understand the psychopathological mechanisms involved in depression.

In traditional theory of psychopathology (i.e., the common cause perspective of mental disorders) psychiatric symptoms are secondary to an underlying common cause [6, 7]. For instance, depression causes low mood, sleep disturbances and suicidality in the same fashion that infection causes fever and pain. Additionally, many studies used standardized scales on depression which sum individual item responses to generate a total score, which implies that depressive symptoms are viewed as interchangeable presentations of the same disorder [8]. However, evidence showed that individual depressive symptoms had different negative outcomes, risk factors and neurological mechanisms [9, 10]. In clinical practice one depressive symptom may predict changes of other symptoms following treatments [11].

In a recently developed theory of psychopathology (i.e., the causal system perspective of mental disorders [12]), the cluster of co-occurring symptoms of depression is secondary to direct symptom-to-symptom relationships, but not a common cause. Accordingly, for example, low mood, anhedonia, and sleep disturbances were not caused by depression. Instead, they could affect each other with their own biological and psychological mechanisms. These symptom-symptom interactions can be estimated using network analysis. Central symptoms within this network exhibit the strongest association with many other symptoms. In addition, since central symptoms may activate other symptoms, they may play a major role in causing the onset of and/or maintaining a syndrome. Thus, targeting central symptoms with biopsychosocial interventions may be more efficient [13]. In the network theory, symptoms can be activated by their neighbor symptoms, or by external factors such as adverse life events, or medical diseases [14]. Network analysis also enables researchers to identify “bridge symptoms” that mediate the transition among different syndromes [15–17].

Network analysis has been used to examine depressive symptoms in certain Western countries [18, 19]. Evidence showed that patterns and clinical features of depression is greatly determined by socioeconomic contexts [20, 21], therefore symptom network structure of depression across different countries and socioeconomic backgrounds should be examined separately. Most studies on network structure of depression were conducted in the West, therefore the findings cannot be generalized in Asian settings including Hong Kong. More importantly, to date, only a paucity of studies utilized the network analysis to examine symptoms and symptom-to-symptom relationships in depression during the COVID-19 pandemic, despite depression having a high prevalence of psychiatric comorbidity in many populations [22]. This gave us the impetus to conduct this study to characterize the network structure of depressive symptoms in a large community adult sample in Hong Kong.

## Methods

### Settings and participants

This large-scale, cross-sectional study was conducted in Hong Kong between 24 March and 20 April 2020 using snowball convenience sampling. The questionnaire was designed using Google form and Qualtrics, and delivered to several online platforms (e.g., WhatsApp, WeChat, Facebook). To be eligible, participants were Hong Kong residents who lived in Hong Kong during the COVID-19 pandemic, aged between 18 and 59 years and were able to read Chinese. This study protocol was approved by the Human Subjects Ethics Sub-committee of the Hong Kong Polytechnic University, Hong Kong (reference number: HSEARS20200227002-01). Participants provided their electronic informed consent prior to participation in this study. Participants were assured of their anonymity and confidentiality, and their rights of withdrawal were respected. Given the sensitive nature of some of the questions, a professional helpline directory was provided to participants.

### Measurements

Depressive symptoms were measured by the Chinese version of the self-report nine-item Patient Health Questionnaire (PHQ-9) [23] that measured a variety of cognitive, emotional, physiological and interpersonal symptoms of depression, such as Anhedonia, Sad Mood, Sleep, Energy, Appetite, Guilt, Concentration, Motor, and Suicide thoughts in the past two weeks [24]. Each item scored from 0 (not at all) to 3 (nearly every day), with a higher score indicating more severe depressive symptoms. The PHQ-9 has been well-validated in the Chinese populations [25–27].

### Network estimation

The mean, standard deviation (SD), skewness and kurtosis of all the PHQ-9 items were calculated. Due to the controversy on the optimal method of model trichotomous items in network analysis [8], the values of all the PHQ-9 items were dichotomized as “0” and “1”, representing the absence and presence of depressive symptoms, respectively. Item values reporting “0” were transformed to absence of the symptom, whereas values of “1, 2, or 3” were transformed to presence of the symptom. The network model was estimated using the Ising model [28].

In the network analysis each individual depressive symptom was defined as “node” and relationships between these symptoms were “edges”. For network visualization, the thickness of edges represented the strength of associations between nodes. The color of the edge indicated the direction of the correlations (e.g., green edges represented positive correlations; red edges represented negative correlations) [29].

The Ising model was used to assess network structures based on binary data [28, 30], which can be conceived as a series of pairwise associations among binary variables, after controlling for the confounding effects of all other associations. This method combined logistic regression with model selection based on a Goodness-of-Fit measure to identify relationships between nodes. To reduce the number of spurious edges and improve the interpretability of networks, the network models were regularized using the enhanced least absolute shrinkage and selection operator (eLASSO) [31]. This algorithm produced a *sparse* network model which became more interpretable than the original one. Model selection was based on the extend Bayesian Information Criterion (EBIC) [32]. The binary network was fitted using the R-package IsingFit 0.3.1 [28].

Three major centrality indices (i.e. Betweenness, Closeness, and Strength) were computed to examine which symptoms were most important in the depressive symptom network [33, 34]. Strength was used to measure the absolute sum of edge weights connected to a node, which indicated the importance of a particular factor. Betweenness was calculated by the frequency of a node lying on all the shortest paths between other nodes, while Closeness referred to the inverse of sum of distance from a node to all other nodes in the network [13]. The R packages “IsingFit” “networktools” and “qgraph” in R program (version 3.6.3) [35] were used to perform the analyses [28, 29].

### Estimation of network accuracy and stability

To assess the robustness of the results, we examined the accuracy and stability of the network model with three procedures [36]. First, the accuracy of edge-weights was estimated by computing confidence intervals (CIs) with non-parametric bootstrapping method [37]. Then, the observations in the data were resampled randomly to create new datasets from which the 95% CIs were calculated. Larger CIs suggested reduced precision in the estimation of the edges, and narrower CIs indicated a more trustworthy network [36].

Second, the correlation stability coefficient (CS-C) was used to assess the stability of centrality indices (i.e. Betweenness, Closeness, and Strength) using subset bootstraps [38]. If centrality indices of nodes did not change significantly after excluding part of the sample in the dataset, the network structure could be considered stable. The CS-C represented the maximum proportion of samples that could be removed, such that with 95% probability the correlation between original centrality indices could reach at least 0.7 [36]. Generally, the CS-C should not be less than 0.25, and preferably above 0.5. Then, the difference between two strength indices was considered significant if 1000-bootstrap 95% non-parametric CIs did not contain zero.

Third, *bootstrapped difference tests* were used to evaluate differences in the network’s properties [36]. This test relied on 95% CIs, to determine if two edge-weights or two node centrality indices significantly differed from one-another. The R package “bootnet” was used to perform the analyses [39].

### Association between symptom mean levels, variability, and centrality index

Spearman’s rank-order correlation was calculated between centrality indexes and the mean PHQ-9 item scores, and between centrality indexes and standard deviation for symptoms [18]. The correlation between centrality indexes and the mean PHQ-9 item scores was used to test whether the most central symptoms are the most severe ones, while the correlation between centrality indexes and standard deviation was used to test whether symptom centrality could be attributed to the items’ differential variability [9].

### Comparison of network characteristics by gender

Following previous studies [18, 19, 40], the differences of network characteristics between male and female participants were examined, using the Network Comparison Test (NCT), a permutation test that assessed the difference between two networks (e.g., male participants vs. female participants) [41]. The NCT was performed on subsamples defined by gender using 1000 permutations as recommended previously [18, 42]. This procedure assessed the global network strength by comparing the absolute sum of all edge weights between the networks. Next, the distributions of edge weights were compared within each network in order to characterize the structure of the network. Finally, the differences in strength for each edge were compared between the two networks after controlling for multiple tests (Holm–Bonferroni correction of *p* values). All the tests were performed with the R-package “NetworkComparisonTest” 2.0.1 [43].

## Results

### Descriptive statistics

A total of 11 072 participants fulfilled the study entry criteria and were included in this study, with 2105 males and 8815 females. Table 1 shows the basic socio-demographic characteristics of the participants. Mean, SD, skewness, and kurtosis, of depressive symptoms measured by the PHQ-9 are shown in Table 2. The mean (SD) of PHQ-9 total score was 0.66 (0.22) after they were transformed into binary variables for the Ising model. The items of Energy and Anhedonia had the highest mean ratings, while the symptoms of Suicide thoughts and Motor problems had the lowest mean ratings.

**Table 1:** Socio-demographic characteristics of the study population (*N* = 11 072).

Variables	*N*	%
Gender ^a^		
Men	2105	19.0
Women	8815	79.6
Married/cohabiting	5933	53.6
Education level ^a^		
Elementary or below ^b^	25	0.2
High school	2668	24.1
College or higher	8244	74.5
Low income ^c^	1289	11.6
Living alone	399	3.6
	Mean	SD
Age (years)	39.07	8.83
PHQ-9 total score	9.60	6.04

*SD* standard deviation, *PHQ-9* the 9-item Patient Health Questionnaire.

^a^ There are missing values, therefore, the total percentage is not equal to 100%.

^b^ Low income = low household’s monthly income (<100 hundred HKD ≈ 1288 dollar).

^c^ Elementary or below = less than 7 years of education.

**Table 2:** Mean, standard deviation, minimum, maximum, skewness, and kurtosis, and frequency of depressive symptoms as measured by the PHQ-9 (*N* = 11 072).

Depressive symptoms	PHQ-9 item	*M*	SD	Min	Max	Skewness	Kurtosis	% (Absence)	% (Presence)
Anhedonia	1	0.86	0.34	0	1	−2.11	2.47	13.7	86.3
Sad Mood	2	0.82	0.39	0	1	−1.64	0.68	18.3	81.7
Sleep	3	0.79	0.41	0	1	−1.4	−0.04	21.3	78.7
Energy	4	0.87	0.33	0	1	−2.23	2.95	12.8	87.2
Appetite	5	0.72	0.45	0	1	−0.98	−1.04	28.0	72.0
Guilt	6	0.56	0.5	0	1	−0.24	−1.94	44.0	56.0
Concentration	7	0.62	0.49	0	1	−0.49	−1.76	38.0	62.0
Motor	8	0.47	0.5	0	1	0.11	−1.99	52.8	47.2
Suicide	9	0.22	0.42	0	1	1.32	−0.26	77.5	22.5

*M* mean, *Min* minimum, *Max* maximum, *PHQ-9* The Patient Health Questionnaire-9, *SD* standard deviation.

### Network structure and centrality measures analysis

Following previous studies [18, 44], item informativeness (i.e., SD of the item) and item redundancy were checked first. We found that no item was poorly informative (i.e., 2.5 SD below the mean level of informativeness [18], *M*_SD_ = 0.43±0.07) and no item was redundant with any other item (i.e., <25% of statistically different correlations). Therefore, all the PHQ-9 items were included in the analyses.

**Fig. 1:**
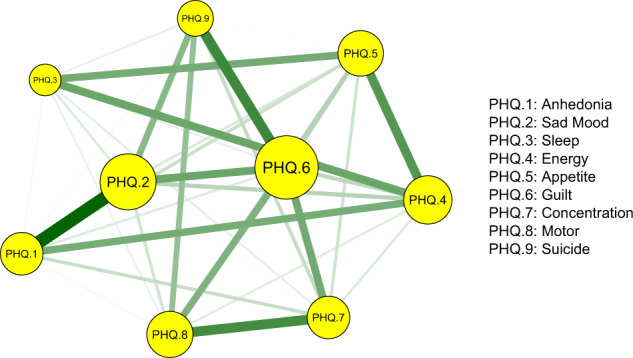
Estimated network model for dichotomized depressive symptoms in the total sample. The network model was estimated using the Ising model.

**Fig. 2:**
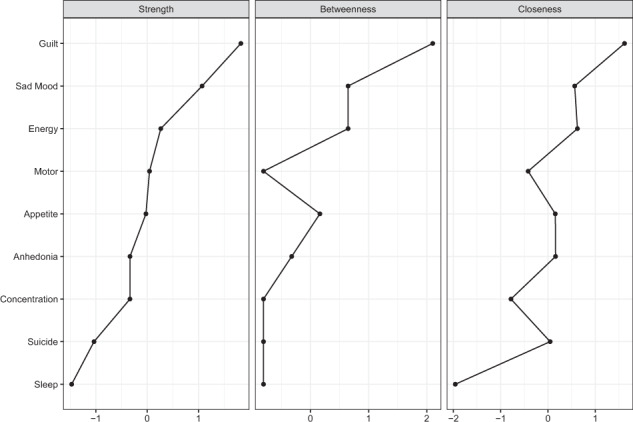
Centrality measures of all symptoms within the network. The figure shows centrality measure (i.e., strength, betweenness, and closeness) of all factors within the network (*z*-scores).

The network of depressive symptoms, as estimated by the Ising model, is shown in Fig. 1. Several nodes were highly connected with the rest of the network, including Guilt (item 6), Sad Mood (item 2) and Energy (item 4). Moreover, there were strong positive correlations between Anhedonia-Sad Mood, Guilty-Suicide, Concentration-Motor, Energy-Appetite, Sad Mood-Guilty, and Sleep-Energy. A weighted adjacency matrix was used to examine the numerical interactions between these symptoms (Supplementary Table 1). Fig. 2 illustrated centrality measures (i.e., strength, betweenness, and closeness) of all the symptoms within the network. The symptom Guilt showed the highest strength, betweenness, and closeness, followed by Sad Mood and Energy.

### Network accuracy and stability

**Fig. 3:**
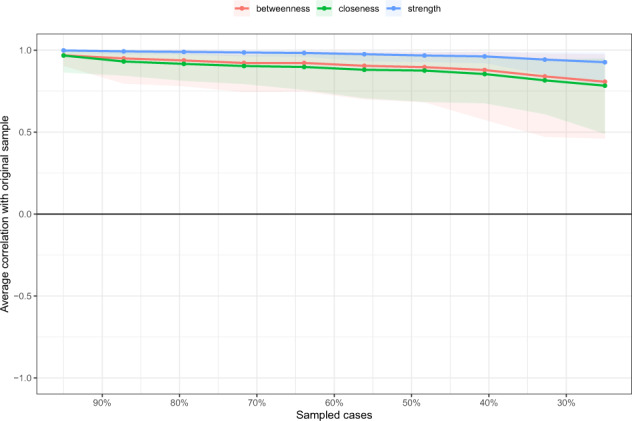
Stability of centrality indices by case dropping subset bootstrap. The *x*-axis represents the percentage of cases of the original sample used at each step. The *y*-axis represents the average of correlations between the centrality indices from the original network and the centrality indices from the networks that were re-estimated after excluding increasing percentages of cases. Each line indicates the correlations among betweenness, closeness, and strength.

The edge weights in the current sample were consistent with the bootstrapped sample, especially the connections with larger weights, indicating that the current network structure was stable (Supplementary Fig. 1). The case-dropping subset bootstrap procedure showed that the values of betweenness, closeness and strength remained stable even after dropping large proportions of the sample (Fig. 3). Although betweenness reported slightly low stability (i.e., CS-C = 0.52), closeness showed higher stability (i.e., CS-C = 0.59) compared to the primary one. In contrast, the strength index in this sample was robust and trustworthy (i.e., CS-C = 0.75; i.e., after dropping up to 75% of the sample, the order of the symptoms in strength was still correlated with the original one (*r* = 0.7)). Hence, we focused on the interpretation of symptom strength based on this network analysis.

**Fig. 4:**
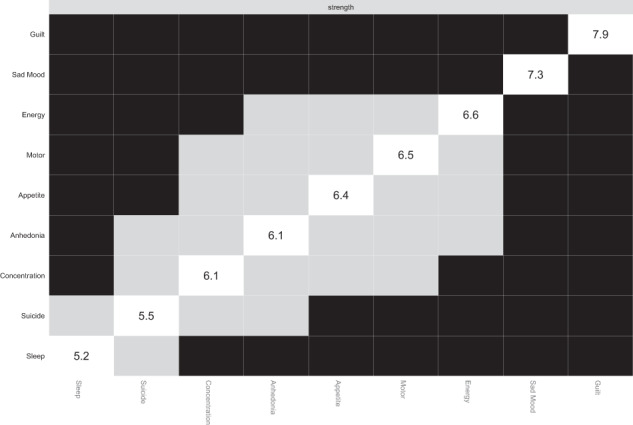
Estimation of node strength difference by bootstrapped difference test. Bootstrapped difference tests between node strength of factors. Gray boxes indicate nodes that do not significantly differ from one-another. Black boxes represent nodes that differ significantly from one another (α = 0.05). White boxes show the values of node strength.

In terms of strength, Guilt, and Sad Mood were statistically stronger compared to other symptoms. Energy, Motor and Appetite appeared stronger than other symptoms in the network (Fig. 4). The bootstrapped difference tests also revealed that a large proportion of the comparisons among edge weights were statistically significant (Supplementary Fig. 2).

### Symptom mean levels, variability, and association with strength centrality index

In the whole sample, depressive symptoms with the highest mean levels were Energy, Anhedonia, Sad Mood and Sleep (Table 2). However, the mean PHQ-9 symptom level was not related to the symptom strength (*r*_*s*_ = 0.15), while symptom standard deviation was also not correlated to the symptom strength (*r*_*s*_ = 0.21, which indicates that high symptom centrality was not related to the mean level of the symptoms and their variability.

### Network and symptom mean levels comparisons between the two genders

**Fig. 5:**
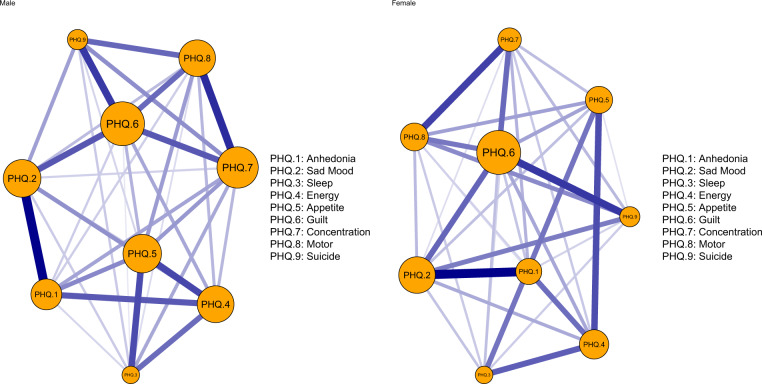
Estimated network models for dichotomized depressive symptoms in male and female participants. Left panel: network structure in male participants (*n* = 2 105); Right panel: network structure in female participants (*n* = 8 815).

We compared the network models and network centrality indices between male (*n* = 2 105) and female participants (*n* = 8 815) (Fig. 5 and Supplementary Fig. 3). There were no significant gender differences in network global strength (males: 3.81 vs. females: 3.77; global strength difference = 0.03, *p* = 0.259), distribution of edge weights (*M* = 0.06, *p* = 0.73) and individual edge weights (all *p* values > 0.05 after Holm-Bonferroni corrections) (Supplementary Fig. 4).

In contrast, there were gender differences in mean levels of certain individual symptoms (Supplementary Table 2). Female participants reported significantly higher mean levels of Sleep problem and Appetite (*P* < 0.01), while male participants reported higher mean levels of Sad Mood, Guilt, Motor, and Suicide thoughts (*P* < 0.01). Similar to the findings obtained in the whole sample, symptom strength was not related to items’ variability in both female (*r*_*s*_ = 0.22) and male (*r*_*s*_ = 0.21) participants. Strength was not correlated to symptoms mean levels in either gender (*r*_*s*_ = 0.12 in female and *r*_*s*_ = 0.25 in male participants), indicating that network symptoms strength and mean levels of symptoms were independent between male and female samples.

### Covariates (age, education level, and marital status)

Previous studies found that age, education level and marital status were associated with the epidemiology and clinical features of depression [20]. Hence, following previous studies [19], the network model and the local structure indexes were re-estimated, after controlling for age, education level, and marital status as covariates. Compared to the original network model, an almost identical network was obtained with respect to the magnitude of edges (*r* = 0.995, 95% CI = [0.991; 0.997], *P* < 0.01), and strength (*r* = 0.984, 95% CI = [0.931; 0.997], *P* < 0.01)) (Supplementary Fig. 5).

## Discussion

To the best of our knowledge, this was the first study to characterize the network structure of depressive symptoms among the general population in Hong Kong. Guilt was the most central symptom in the network of depression, followed by Sad Mood and Energy symptoms that could maintain or trigger the remaining depressive symptoms in this sample.

Guilt was the most central symptom in Hong Kong residents during the COVID-19 pandemic. Guilt is an interpersonally driven emotion arising from the belief that an individual has hurt others. Due to this belief, guilt is often accompanied by regret, remorse, or worries over a transgression leading to depressive states [45]. Guilt may manifest as self-blame, worthlessness, powerlessness, inferiority [46], hopelessness and helplessness [47], which could lead to development of depressive symptoms [46]. Energy is another central symptom in this study, which is consistent with the findings of a previous drug trial of duloxetine, with energy improvement as a major outcome measure [48]. A previous study [49] compared gender differences in the associations between individual symptoms of major depression and other psychiatric disorders with network analysis using the data extracted from the 2011 and 2016 Korean Epidemiologic Catchment Area Study (n = 907), and found that the global strength did not differ between genders, which is consistent with our findings.

Sad Mood is another central depressive symptom in the network of Hong Kong residents, which is consistent with the Beard et al.’s finding [50] that ‘sad mood’ and ‘too much worry’ were the most central to the network of depressive symptoms as measured by the PHQ-9 and the 7-item Generalized Anxiety Disorder Scale (GAD-7). However, Beard et al.’s study used network analysis to investigate the depression and anxiety symptom relationships on psychiatric inpatients in the US, whilst our study investigated depressive symptoms in a community sample. Despite different study samples, sad mood is the hallmark symptom required for meeting the diagnosis of major depression [51]. Additionally, our findings are similar to another study using network model with the PHQ-9 in the German general population [52], in which sad mood, energy loss and guilt were central depressive symptoms. In another study using network analysis on depression and anxiety symptom relations as measured by the PHQ-9 and GAD-7 in a psychiatric sample [50], “sad mood” was the most central symptom, followed by certain anxiety symptoms such as “too much worry”, “unable to control worry”, and “unable to relax”; furthermore, the most central symptoms in the depression community were low energy, anhedonia, and guilt, which partly supports our findings. In another study on the changes in network centrality of psychiatric symptoms as measured by the GAD-7 and PHQ-9 between the COVID-19 outbreak and after peak, “loss of energy” played an important role in the network model [42].

The NCT did not reveal significant gender differences in the network structures of depressive symptoms, which is consistent with the findings of previous network analyses [18, 19, 40], but does not directly support the notion that there are different clinical features between the two genders [53–57]. Epidemiological studies found significant gender differences in depression prevalence [53], with higher rates of depression in women. A systematic review revealed a female preponderance of 1.5–3 times higher depression rates [55]. For clinical features of depression, the STAR*D study showed that appetite and weight changes, anergia, psychomotor agitation and sympathetic arousal symptoms were usually over-represented in women [58]. In another study of twins with depression, female twins usually experienced more severe fatigue, hypersomnia and psychomotor retardation, while male twins experienced more severe insomnia and agitation [59]. However, in the current network analysis, no significant gender differences in the network structures of depressive symptoms were found. The different results between this and the abovementioned studies could be explained by several reasons. First, most studies on epidemiology and clinical features of depression were based on total score of standardized scales on depression such as the PHQ-9, which however has the potential to obscure the important differences between individual symptoms and the relationships among symptoms [50]. In contrast, the network approach assesses interactions between individual symptoms [6]. Second, considering that disproportional gender ratio (Male=2 105 VS. Female=8 815) in the current sample, the findings on gender differences in this study are preliminary. Finally, it is possible that the gender differences in network analysis may be partly masked by the negative impact of the pandemic on mental health, as both genders may be equally affected by fears about the pandemic and related consequences and public health measures such as lockdown.

In the past years Hong Kong has been confronted by repeated co-occurrence of population-level stressors including social unrest (e.g., the Occupy Central Movement (Umbrella Movement) in 2014; anti-extradition bill in 2019) and public health crises (e.g., COVID-19 pandemic in 2019), all of which were associated with increased risk of mental problems including depression. For instance, some studies [60, 61] examined the association between the Occupy Central Movement and population mental distress using anonymous random population-based telephone survey, and results showed that young age (of those who did not participate in the movement), worry about safety, negative emotional responses to media reports and local political situations, conflicts with peers about this movement were significantly associated with mental distress.

In 2019, a series of local protests against the extradition bill progressed from initial political protests to violent protests in Hong Kong, ended up with thousands of arrests [62]. This social unrest has brought about tremendous mental distress to Hong Kong residents [63, 64]. The subsequent outbreak of COVID-19 in early 2020 has made the matters even worse as this pandemic has led to widespread panic in Hong Kong. The rapid transmission of this novel coronavirus locally and globally, in the absence of effective treatment/vaccine, alongside insufficient personal protective equipment (e.g., face-masks) and conflictual messages by the local government on quarantine, social distancing and contingent public health preventive measures (closure of major theme parks, entertainment amenities, sports gymnasiums, suspension of face-to-face teaching/learning in all educational institutions) have reminded the lay public their traumatic memories during the 2003 SARS epidemic [65].

At the time of report, Hong Kong is in the fourth wave of the COVID-19 pandemic. This long-haul battle combating against the pandemic, alongside with recurrent political instability may cause unease, anxiety and depression in the general population [66]. For example, Wong et al [67]. examined the impact of repeated exposure to social unrest-related traumatic events and COVID-19, pandemic-related events on Hong Kong residents and found that rumination, stressful life events and pandemic-related events were significant predictors (all *P* < 0.001) of depression. All subtypes of ruminations were significant mediators [67], which is consistent with our finding that guilt is the key central depressive symptom in this study.

Due to the recent social unrest and public health crises that happened in Hong Kong, rumination (guilt) is the central depressive symptoms in this network analysis. Nevertheless, previous studies predominantly focused on depressive rumination [68] but rarely examined rumination relating to external societal and environmental situations, although such rumination may have a substantial long-term impact on population mental health leading to increased psychiatric morbidities (particularly depression) and global disease burden. A 10-year cohort study [63] on mental health burden and associated factors of depression and posttraumatic stress disorder (PTSD) found that the weighted prevalence of depression among Hong Kong adults was 1.9% (95% CI: 1.6–2.1%) at baseline (between 2009 and 2014). In contrast, the weighted prevalence of depressive symptoms increased to 37.4% (95% CI: 35.1–39.7%), with 11.2% (95% CI: 9.8–12.7%) having probable depression in 2019–2020.

The strengths of this study include the large sample size and the homogenous study sample. However, several implications needed to be addressed. First, this is a cross-sectional study, therefore the causality and the dynamic relationships between variables cannot be estimated. Second, this study was conducted in a community adult sample, hence, the findings cannot be generalized to special populations such as adolescents, the elderly, and patients with major depression. Third, since patterns and clinical features of depression are greatly determined by socioeconomic contexts [20, 21, 64], the structure of depressive symptoms in settings with different socioeconomic and cultural contexts should be examined separately. Fourth, depressive symptoms were assessed by PHQ-9 rather than clinical interview; therefore, atypical features of depression could not be identified, which may bias the results to an uncertain extent. Finally, the possibility that some participants suffered from depressive disorders could not be excluded, which may bias the findings to an uncertain extent.

In conclusion, this network analysis revealed that Guilt, Sad Mood and Energy were the most central symptoms of depression in this study. These three central symptoms constitute the “backbone” that sustained the depressive symptom structure among Hong Kong residents during the COVID-19 pandemic, which provided a unique opportunity to understand the interactions between prolonged social unrest and recurrent public health crisis may contribute to depressive symptoms among Hong Kong residents amidst COVID-19 pandemic. Timely mental health treatment to reduce rumination, such as cognitive behavioral therapy (CBT), was crucial in the prevention of further exacerbation of depressive symptoms among Hong Kong residents especially during the pandemic era and beyond.

## Supplementary information

Supplemental material

## Data Availability

Analytic code for this work is available upon request.
