# Supplementary material for: Network analysis of depressive symptoms in Hong Kong residents during the COVID-19 pandemic
Source: Transl Psychiatry. 2021 Sep 6;11:460. doi: 10.1038/s41398-021-01543-z (PMC8419676; doi:10.1038/s41398-021-01543-z)
Supplement: Supplementary file 1 — Supplemental material [file 41398_2021_1543_MOESM1_ESM.docx]

**Supplementary Information**

**Supplementary figure and table legends**

Supplementary Table 1. Weighted adjacency matrix

Supplementary Figure 1. Bootstrapped confidence intervals of edge weights

Supplementary Figure 2. Estimation of edge weight difference by bootstrapped difference test

Supplementary Figure 3. Comparison of network centrality indices between male and female participants

Supplementary Figure 4. Comparison of network properties between male and female participants

Supplementary Figure 5. Estimated network model for dichotomized depressive symptoms in the whole sample after controlling for age, education and marital status

Supplementary Table 2. Mean, standard deviation, skewness, and kurtosis, and frequency of depressive symptoms as measured using the PHQ-9 by gender

Information of the International Research Collaboration on COVID-19

**Supplementary Table 1. Weighted adjacency matrix**

|  | Anhedonia | Sad Mood | Sleep | Energy | Appetite | Guilt | Concentration | Motor | Suicide |
| --- | --- | --- | --- | --- | --- | --- | --- | --- | --- |
| Anhedonia | 0.00 | 2.14 | 0.32 | 1.27 | 0.64 | 0.50 | 0.66 | 0.31 | 0.30 |
| Sad Mood | **2.14** | 0.00 | 0.50 | 0.78 | 0.52 | 1.35 | 0.29 | 0.59 | 1.13 |
| Sleep | 0.32 | 0.50 | 0.00 | 1.35 | 1.28 | 0.44 | 0.44 | 0.41 | 0.43 |
| Energy | 1.27 | 0.78 | **1.35** | 0.00 | 1.52 | 0.74 | 0.51 | 0.46 | 0.00 |
| Appetite | 0.64 | 0.52 | 1.28 | **1.52** | 0.00 | 0.73 | 0.61 | 0.80 | 0.28 |
| Guilt | 0.50 | **1.35** | 0.44 | 0.74 | 0.73 | 0.00 | 1.30 | 1.19 | 1.70 |
| Concentration | 0.66 | 0.29 | 0.44 | 0.51 | 0.61 | 1.30 | 0.00 | 1.64 | 0.67 |
| Motor | 0.31 | 0.59 | 0.41 | 0.46 | 0.80 | 1.19 | **1.64** | 0.00 | 1.03 |
| Suicide | 0.30 | 1.13 | 0.43 | 0.00 | 0.28 | **1.70** | 0.67 | 1.03 | 0.00 |

Weighted adjacency matrix based on factors to represent the weight of direct edges between node.


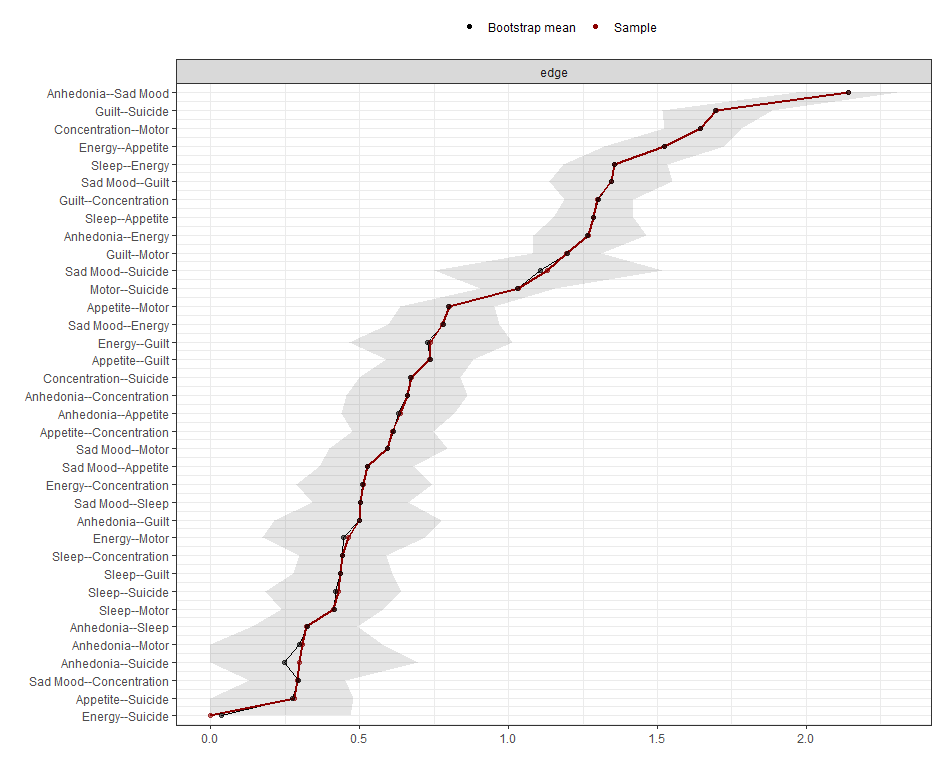


**Supplementary Figure 1. Bootstrapped confidence intervals of edge weights.** The black dots indicate the values of each edge weight, ordered from the highest to the lowest value. The gray area represents the 95% Confidence Intervals of edge weights, estimated with the non-parametric bootstrap procedure (Bootnet package). Wide intervals indicate lower stability and narrow intervals indicate higher stability.


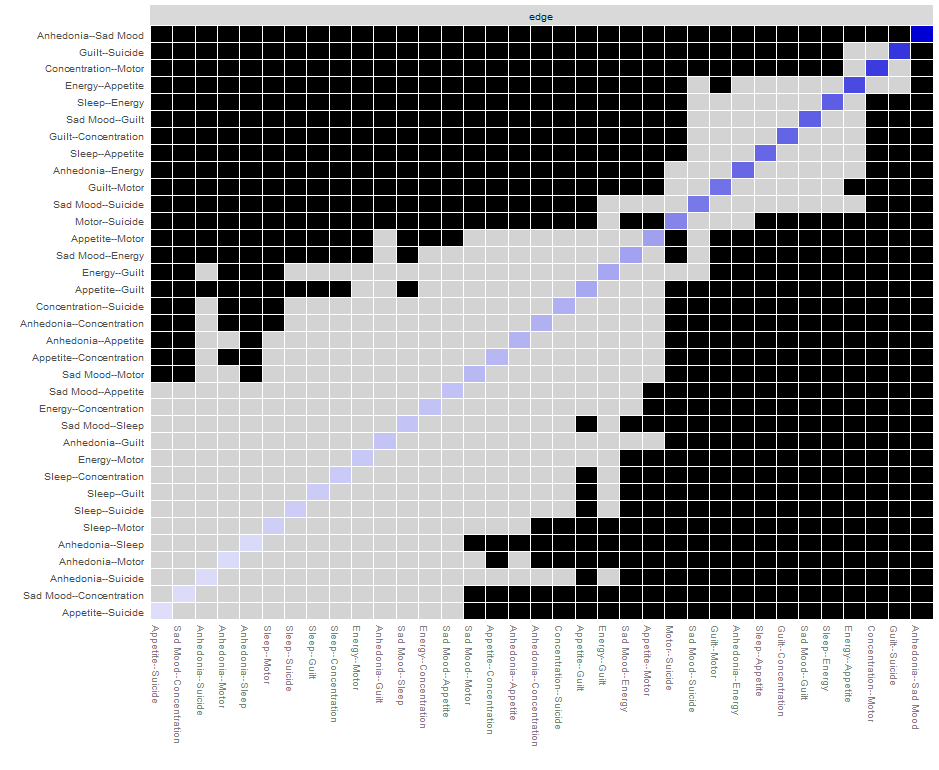


**Supplementary Figure 2. Estimation of edge weight difference by bootstrapped difference test.** Bootstrapped difference tests between edge weights in the network. Gray boxes indicate edges that do not significantly differ from one-another. Black boxes represent edges with significant difference from one another (α = 0.05). Blue boxes in the edge-weight plot indicate positive correlations.


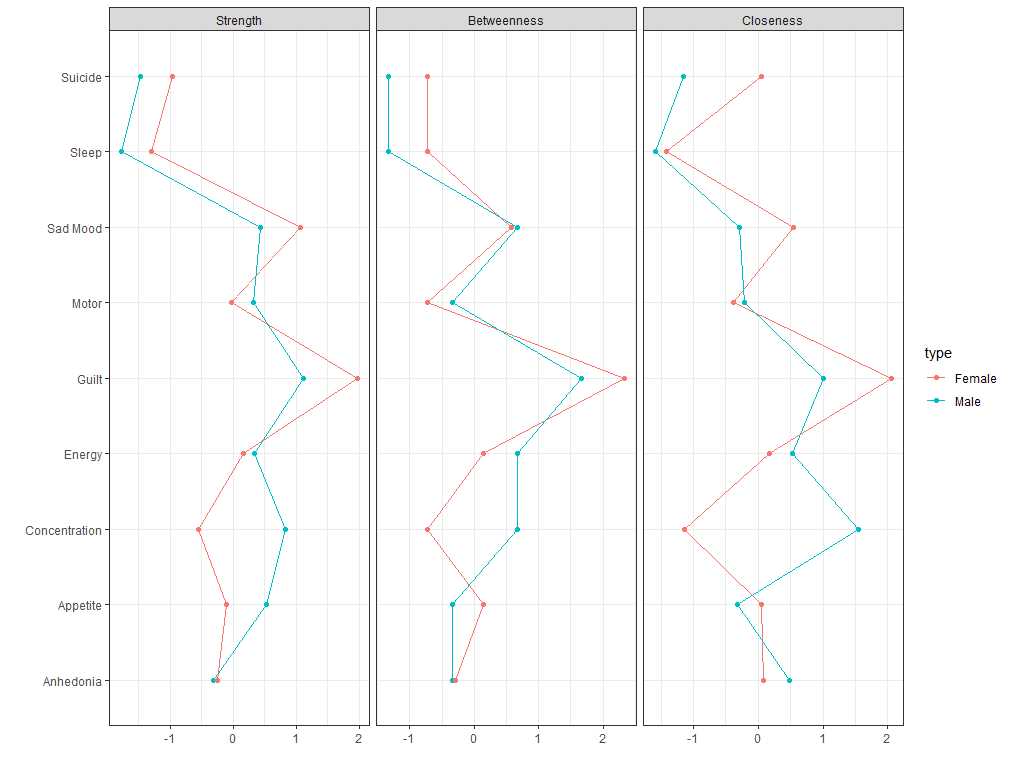


**Supplementary Figure 3.** Comparison of network centrality indices between male and female participants.


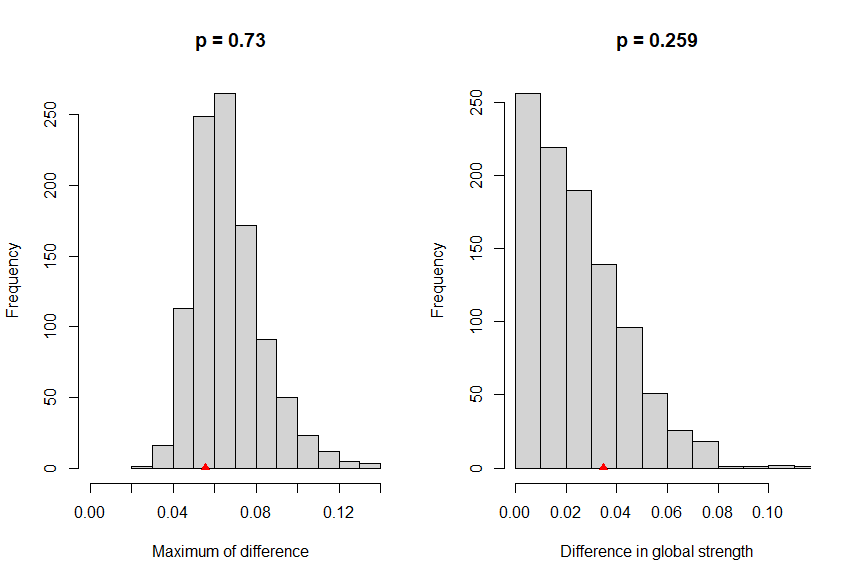


**Supplementary Figure 4. Comparison of network properties between male and female participants.**

The Network Comparison Test (NCT) is a permutation test to investigate invariance in different network characteristics.

Left Panel: Plot of bootstrap value of the maximum difference in any of the edge weights (1000 permutations), with no significant difference (M=0.06, p=0.73).

Right Panel: Plot of bootstrap value of the difference in network global strength, with significant difference (network strength among male participants: 3.81; among female participants: 3.77; S: 0.03, p=0.259).

Invariance in edges weights was examined using the permutation test, generating sets of p values for each edge-edge comparison. Holm-Bonferroni corrected p values were all >0.05 indicating absence of significant differences.

**Supplementary Figure 5. Estimated network model for dichotomized depressive symptoms in the whole sample after controlling for age, education and marital status**


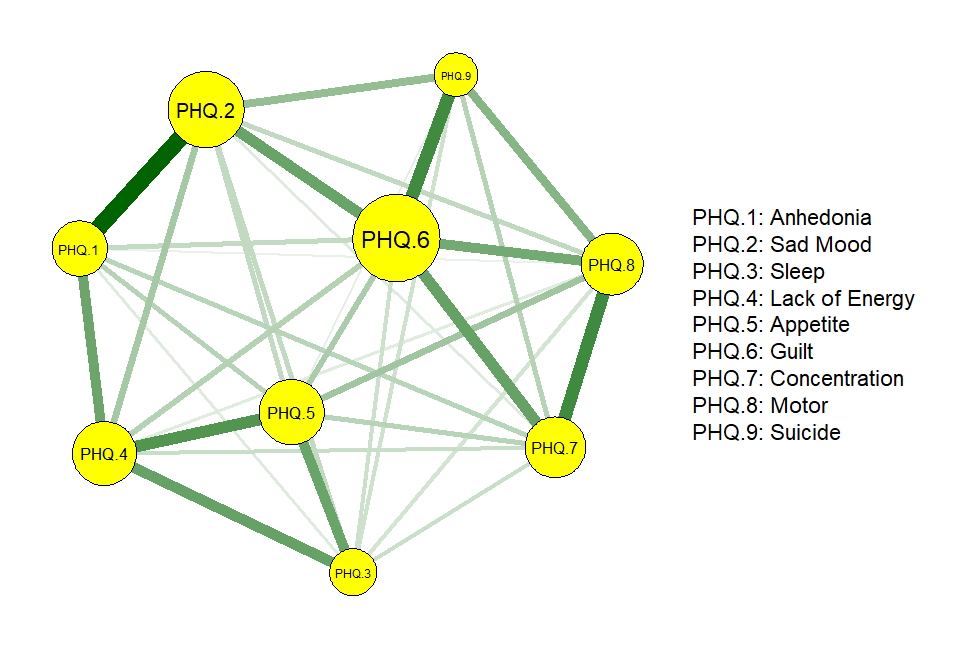


**Supplementary Table 2.** Mean, standard deviation, skewness, and kurtosis, and frequency of depressive symptoms as measured using the PHQ-9 by gender.

|  | Male (n=2,105) | | | | | | Female (n=8,815) | | | | | |  |  |
| --- | --- | --- | --- | --- | --- | --- | --- | --- | --- | --- | --- | --- | --- | --- |
| Depressive symptoms | M | SD | Skewness | Kurtosis | % Absence | % Presence | M | SD | Skewness | Kurtosis | % Absence | % Presence | t-test | P |
| Anhedonia | 0.86 | 0.34 | -2.13 | 2.54 | 13.5 | 86.5 | 0.86 | 0.34 | -2.11 | 2.47 | 13.7 | 86.3 | 0.16 | 0.88 |
| Sad Mood | 0.82 | 0.38 | -1.68 | 0.81 | 17.9 | 82.1 | 0.82 | 0.39 | -1.63 | 0.65 | 18.4 | 81.6 | 0.62 | **<0.01** |
| Sleep | 0.75 | 0.43 | -1.14 | -0.69 | 25.2 | 74.8 | 0.80 | 0.40 | -1.47 | 0.16 | 20.4 | 79.6 | 4.82 | **<0.01** |
| Energy | 0.86 | 0.35 | -2.08 | 2.32 | 14.0 | 86.0 | 0.88 | 0.33 | -2.27 | 3.15 | 12.5 | 87.5 | 1.82 | 0.07 |
| Appetite | 0.69 | 0.46 | -0.8 | -1.37 | 31.5 | 68.5 | 0.73 | 0.45 | -1.02 | -0.95 | 27.2 | 72.8 | 3.94 | **<0.01** |
| Guilt | 0.61 | 0.49 | -0.45 | -1.8 | 39.0 | 61.0 | 0.55 | 0.50 | -0.19 | -1.96 | 45.2 | 54.8 | 5.15 | **<0.01** |
| Concentration | 0.63 | 0.48 | -0.54 | -1.71 | 37.0 | 63.0 | 0.62 | 0.49 | -0.48 | -1.77 | 38.3 | 61.7 | 1.10 | 0.27 |
| Motor | 0.51 | 0.50 | -0.05 | -2 | 48.7 | 51.3 | 0.46 | 0.50 | 0.15 | -1.98 | 53.8 | 46.2 | 4.16 | **<0.01** |
| Suicide | 0.27 | 0.44 | 1.06 | -0.87 | 73.5 | 26.5 | 0.21 | 0.41 | 1.39 | -0.07 | 78.5 | 21.5 | 5.00 | **<0.01** |

Note: M: Mean; PHQ-9: The Patient Health Questionnaire-9; SD: Standard Deviation.

**The International Research Collaboration on COVID-19^#^**

Prof. Lorna Kwai Ping SUEN

BN, MPH, PhD, RN

School of Nursing, Tung Wah College, Hong Kong SAR.

Email: [lornasuen@twc.edu.hk](mailto:lornasuen@twc.edu.hk)

ORCID: 0000-0002-0126-6674

Ms. Shun CHAN

BN, RN

Squina International Centre for Infection Control, School of Nursing, The Hong Kong Polytechnic University, Hong Kong SAR.

Email: [csschan2017@gmail.com](mailto:csschan2017@gmail.com)

Ms. Hilda Sze Wing HO

MA, MPH

Department of Psychology, York University, Canada.

Email: [hildaho@yorku.ca](mailto:hildaho@yorku.ca)

ORCID: 0000-0002-7966-4285

Dr. Kin Bong Hubert LAM

PhD

Nuffield Department of Population Health, University of Oxford, United Kingdom.

Email: [hubert.lam@ndph.ox.ac.uk](mailto:hubert.lam@ndph.ox.ac.uk)

ORCID: 0000-0003-1228-3362

Dr. Emma Yun-zhi HUANG

DPH, MPH

Department of Social Worker, Zhongshan Polytechnic, Guangdong, China.E-mail: [huangyunzhiemma@sina.com](mailto:huangyunzhiemma@sina.com)

ORCiD: 0000-0001-5967-2731

Prof. Ying XIAO

PhD

Professor

Faculty of Medicine, Macau University of Science and Technology, Macao.

Email: [yxiao@must.edu.mo](mailto:yxiao@must.edu.mo)

Dr. Fernanda Maria Vieira Pereira-Ávila

PhD, RN

Fluminense Federal University, Rio das Ostras, Brazil.

Email: [fernandamvp@id.uff.br](mailto:fernandamvp@id.uff.br)

ORCiD: 0000-0003-1060-6754

Prof. Elucir GIR

PhD, RN, Full Professor

University of São Paulo, School of Nursing at Ribeirão Preto, Brazil.

Email: [egir@eerp.usp.br](mailto:egir@eerp.usp.br)

ORCID: 0000-0002-3757-4900

Dr. Menevse YILDIRIM

PhD

Department of Nursing Management, Fethiye Faculty of Health Sciences, Muğla Sıtkı Kocman University, Mugla, Turkey.

Email: [menevseyildirim@mu.edu.tr](mailto:menevseyildirim@mu.edu.tr); [menevsesamur@gmail.com](mailto:menevsesamur@gmail.com)

ORCID: 0000-0001-6033-6196

Prof. Seyda SEREN INTEPELER

PhD

Department of Nursing Management, Faculty of Nursing, Dokuz Eylul University, Izmir, Turkey.

Email: [seydaseren@gmail.com](mailto:seydaseren@gmail.com)

ORCID: 0000-0001-8615-9765

Dr. Tella LANTTA

RN, PhD

Department of Nursing Science, University of Turku, Finland.

Email: [tella.lantta@utu.fi](mailto:tella.lantta@utu.fi)

ORCID: 0000-0001-7715-7573

Dr. Kyungmi LEE

PhD, RN

Samsung Medical Center, Seoul, Korea

Email: [kyungmi79.lee@samsung.com](mailto:kyungmi79.lee@samsung.com)

ORCID: 0000-0002-0381-0071

Dr. Nayeon SHIN

PhD

CHA University, Bundang CHA Medical Center, Seongnam, Korea.

E mail: nabong78@hanmail.net

ORCID: 0000-0002-3994-5867

Mr. Laurence Lloyd PARIAL

MSc

School of Nursing, The Hong Kong Polytechnic University

Email: [laurence.parial@connect.polyu.hk](mailto:laurence.parial@connect.polyu.hk)

ORCID: 0000-0003-3069-0009

Mr. Tor Michael Rossing

MA

SAG Flowmedik Oy, Helsinki, Finland

Email: [michael.rossing@flowmedik.com](mailto:michael.rossing@flowmedik.com)

Ms. Ching Yuk Hon

MSc, BN, RN

School of Nursing, The Hong Kong Polytechnic University

Email: [ching-yuk.hon@connect.polyu.hk](mailto:ching-yuk.hon@connect.polyu.hk)

Ms. Merissa Tsang

MSc

Agape Acupuncture Clinic, San Mateo, California, USA.

Email: [merissa.tsang@gmail.com](mailto:merissa.tsang@gmail.com)

Ms. Jessica P. Braz POEYS

BSN, RN

Westways Staffing Inc., Austin, USA.

Email: [jessicapb@hotmail.com](mailto:jessicapb@hotmail.com)

ORCID: 0000-0002-6431-6288

Mr. Tommy Kwan Hin FONG

BSc, MPsyMed

School of Nursing, The Hong Kong Polytechnic University, Hong Kong SAR.

Email: [kfonguos@gmail.com](mailto:kfonguos@gmail.com)
